# Enhancement patterns of adrenal nodules on magnetic resonance imaging

**DOI:** 10.1590/S1677-5538.IBJU.2021.0472

**Published:** 2022-01-11

**Authors:** Fernando Ide Yamauchi, George Ramos Lemos, André Dubinco, Omir Antunes Paiva, Thais Caldara Mussi, Ronaldo Hueb Baroni

**Affiliations:** 1 Hospital Israelita Albert Einstein Departamento de Radiologia São Paulo SP Brasil Departamento de Radiologia, Hospital Israelita Albert Einstein, São Paulo, SP, Brasil

**Keywords:** Magnetic Resonance Imaging, Adrenal Glands, Carcinoma

## Abstract

**Objective:**

To compare enhancement patterns of typical adrenal adenomas, lipid-poor adenomas, and non-adenomas on magnetic resonance imaging (MRI).

**Materials and Methods:**

Evaluation of adrenal nodules larger than 1.0 cm, with at least 2-year follow-up, evaluated on MRI in January 2007 and December 2016. Two different protocols were included - upper abdomen MRI (delayed phase after 3 minutes) and abdomen and pelvis MRI (delayed phase after 7 minutes) – and nodules were divided in typical adenomas (characterized on out-of-phase MRI sequence), lipid-poor adenomas (based on follow-up imaging stability) and non-adenomas (based on pathological finding or follow-up imaging). T2-weighted and enhancement features were analyzed (absolute and relative washout and enhancement curve pattern), similarly to classic computed tomography equations.

**Results:**

Final cohort was composed of 123 nodules in 116 patients (mean diameter of 1.8 cm and mean follow up time of 4 years and 3 months). Of them, 98 (79%) nodules had features of typical adenomas by quantitative chemical shift imaging, and demonstrated type 3 curve pattern in 77%, mean absolute and relative washout of 29% and 16%, respectively. Size, oncologic history and T2-weighted features showed statistically significant differences among groups. Also, a threshold greater than 11.75% for absolute washout on MRI achieved sensitivity of 71.4% and specificity of 70.0%, in differentiating typical adenomas from non-adenomas.

**Conclusion:**

Calculating absolute washout of adrenal nodules on MRI may help identifying proportion of non-adenomas.

## INTRODUCTION

Incidental nodules in adrenal glands are common findings in radiology daily practice, and identified in up to 5% of computed tomography (CT) exams in patients who are asymptomatic or present symptoms not related to the adrenal gland (1, 2). The incidence of adrenal nodules further increases to 9-13% in patients submitted to imaging examinations for a known malignancy, but only 26-36% of such lesions are metastatic. The vast majority of incidental adrenal lesions are benign non-functioning adenomas, even in patients with known extra-adrenal malignancy (3). However, lesions of concern such as metastasis, carcinomas and pheochromocytoma may also present as incidental findings, and differentiating these lesions on imaging exams is crucial.

Magnetic resonance imaging (MRI) has high accuracy in differentiating benign and malign nodules, especially when evaluating chemical shift imaging (CSI). The majority of adrenal lesions contain high intracytoplasmic lipid-to-water ratio, and according to Weiss, are more likely to be benign adrenocortical neoplasms (4, 5).

The following characteristics are explored on imaging exams and are well established in the literature: measurement of CT density (<10 Hounsfield Units) (6) and signal drop on out-of-phase chemical shift sequence on MRI (7). Lesions that own these strict imaging criteria may presumably be considered lipid-rich adenomas, with no need of follow-up or more aggressive procedures.

Therefore, the majority of adrenal lesions are benign in nature (particularly adenomas), and it is very important to accurately differentiate them from malignant lesions, which will need an interventional procedure. Lesions that do meet the fat content criterion are considered indeterminate and can be further evaluated by their enhancement pattern. Caoili et al. (8) first described a particular enhancement pattern of adenomas on CT, characterized by rapid enhancement followed by rapid washout on delayed phases, as opposed to the majority of malignant lesions. Indeed, the use of enhancement pattern on MRI for adrenal lesions is not well established in clinical practice, unlike CT.

There are few reports evaluating enhancement pattern of indeterminate adrenal lesions on MRI, essentially washout patterns. Few articles reported enhancement of adrenal nodules on MRI, but they included small samples, evaluated only a single characteristic, did not include pheochromocytomas or had short follow-up periods (9–14). Studies with larger samples, including medullar adrenal lesions and with longer follow-up periods, would be important to corroborate the use of enhancement pattern on MRI to evaluate indeterminate adrenal lesions.

Therefore, the objective of this study was to compare enhancement patterns of typical adrenal adenomas, lipid-poor adenomas and non-adenomas on MRI.

## MATERIALS AND METHODS

This is a retrospective study approved by the Institutional Review Board, in which the informed consent was waived (CAAE number 81443117.5.0000.0071). A search for adrenal nodules on MRI was performed on the electronic medical records of the organization, for the period of January 2007 to December 2016.

Inclusion criteria were adrenal nodules with typical findings of adenoma or any adrenal nodule with at least 2-year follow-up. Exclusion criteria were imaging follow-up less than 2 years, lesions characterized as cysts or myelolipomas on MRI, lesions measuring less than 1.0 cm, studies with incomplete or inappropriate protocols.

The diagnosis of adenoma was based on: presence of intracellular fat in an homogeneous adrenal nodule by quantitative chemical shift technique of at least 16.5% of signal drop in out-of-phase sequence (7) or by the temporal stability of an homogeneous lesion for at least two years. The diagnosis of non-adenoma lesions was made by pathology result after surgical resection: newly-developed or growth of lesion in an oncologic patient, which was considered metastasis or newly-developed heterogeneous lesions.

MRI examinations were performed using several different 1.5 or 3 Tesla scanners. Adrenal lesions were analyzed in two different magnetic resonance protocols: upper abdomen MRI and abdomen and pelvis MRI. Protocol details are summarized on supplementary table. On upper abdomen protocol, delayed phase was acquired 3 minutes after administration of contrast medium. On abdomen and pelvis protocol, this delayed phase was acquired with a mean of 7 minutes (range from 4 minutes to 12 minutes), due to post-contrast sequence on the pelvis. The contrast dose used in the studies was 0.2mL/kg body weight, with an injection rate of 2mL/s.

One radiologist (board certified radiology with one-year experience in abdominal imaging) reviewed all medical records and MRI exams. The study coordinator anonymized the exams, which were evaluated using a PACS (Picture Archiving and Communication System) workstation (KODAK/Carestream; Carestream Health, Rochester, New York, USA). First, the signal intensity on T2-weighted imaging was compared to the normal gland parenchyma (homogeneous vs. heterogeneous and hyposignal vs. hypersignal). Then, regions of interest (ROI) were placed on adrenal nodules on axial plane in at least one half to two-thirds of the diameter, in the following sequences: in and out-phase, pre-contrast, portal phase and equilibrium/delayed phase, and signal intensity was recorded for each sequence.

Three enhancement features were evaluated: absolute washout, relative washout and enhancement curve pattern. The first two were calculated similarly to classic CT equations (8): (1) absolute washout = (portal phase signal-delayed phase signal) / (portal phase signal-unenhanced signal); (2) relative washout = (portal phase signal-delayed phase signal) / (portal phase signal).

The enhancement curve pattern was analyzed according to the standards used in breast MRI exams (15), comparing signal intensity on portal and delayed phases. Type 1 curve (or progressive) when there is progressive enhancement from portal to delayed phase and more than 10% signal increase occurred. Type 2 curve (or plateau) when signal intensity remained between-10% and+10% from portal to delayed phase. And type 3 (or regressive) when more than 10% signal decrease occurred from portal to delayed phase.

Statistics were performed using R studio (Version 1.0.153). ANOVA test was employed to evaluate the variable age. To evaluate sex, oncologic history and signal on T2-weighted imaging, the chi-square test was used. And finally, to evaluate size, absolute washout and relative washout the Kruskal-Wallis test was used.

## RESULTS

The initial search resulted in 708 adrenal nodules during the experimental period. A total of 585 nodules were excluded for the following reasons: lesions with less than 2-year follow-up (n=518); myelolipomas (n=18); cysts (n=8); lesions smaller than 1.0cm (n=13), incomplete exam protocols (n=27), and retroperitoneal lesion confirmed as non-adrenal on surgery (n=1). The final cohort was composed of 123 nodules in 116 patients (50 men and 66 women), mean age of 57 years (range from 22 to 88 years), and mean follow-up of 4 years and 3 months (range from 24 to 95 months). Mean nodule diameter was 1.8cm (ranging from 1.0 to 12.6cm); in that, 44 were in the right adrenal gland and 79 in the left.

Ninety-eight of 123 (79%) nodules had typical features of adenomas by quantitative chemical shift imaging, 15 (12%) nodules were presumably considered as lipid-poor adenomas based on stability and 10 (9%) lesions were considered as non-adenoma. The non-adenoma lesions comprised pheochromocytomas (n=3), adrenal carcinomas (n=2), metastasis of melanoma (n=1), collision tumor (n=2), mesenchymal neoplasm (n=1), and neoplasm of peripheral nerve sheath (n=1). Characteristics of lipid poor adenomas and non-adenomas are summarized on Tables 1 and 2, respectively.

**Table 1:** Size, enhancement pattern and protocol performed on magnetic resonance imaging of the evaluated lipid poor adrenal adenomas (N= 15).

Size (cm)	Curve Pattern	Absolute washout (%)	Relative Washout (%)	Protocol (delayed phase time)
1.0	3	42	18	Superior (3′00″)
1.0	3	25	12	Superior (3′00″)
1.0	2	25	7	Superior (3′00″)
1.1	3	71	35	Total (6′23″)
1.2	2	0	0	Superior (3′00″)
1.2	3	34	15	Total (7′24″)
1.2	3	30	13	Total (5′37″)
1.3	2	0	0	Superior (3′00″)
1.4	1	0	0	Total (10′24″)
1.6	3	33	20	Total (5′54″)
1.6	3	31	16	Total (6′28″)
1.8	3	27	11	Superior (3′00″)
2.0	2	0	0	Superior (3′00″)
2.0	1	0	0	Superior (3′00″)
2.5	1	0	0	Total (7′32″)

**Table 2:** Final diagnosis, size, enhancement pattern and protocol performed on magnetic resonance imaging of the evaluated lipid poor adrenal adenomas (N= 10).

Diagnosis	Size (cm)	Curve Pattern Type	Absolute Washout	Relative Washout	Protocol (delayed phase time)	Diagnosis
Metastasis (melanoma)	1.5	2	7	3	Superior (3′00″)	ND
Pheochromocytoma	2.0	3	36	15	Total (6′44″)	AP
Mesenchymal neoplasm	3.5	3	27	13	Total (6′31″)	AP
Collision metastasis (bladder)	3.5	2	9	5	Total (5′04″)	ND
Pheochromocytoma	3.5	1	0	0	Total (12′30″)	AP
Collision cyst into adenoma	4,0	3	25	15	Superior (3′00″)	IM
Pheochromocytoma	4.7	2	6	1	Superior (3′00″)	AP
Carcinoma	6.5	3	21	12	Total (7′41″)	AP
Peripheral nerve sheath neoplasm	10.0	1	0	0	Superior (3′00″)	AP
Carcinoma	12.6	2	22	9	Total (7′56″)	AP

AP = anatomopathological; ND = newly developed; IM = imaging

**Figure 1:**
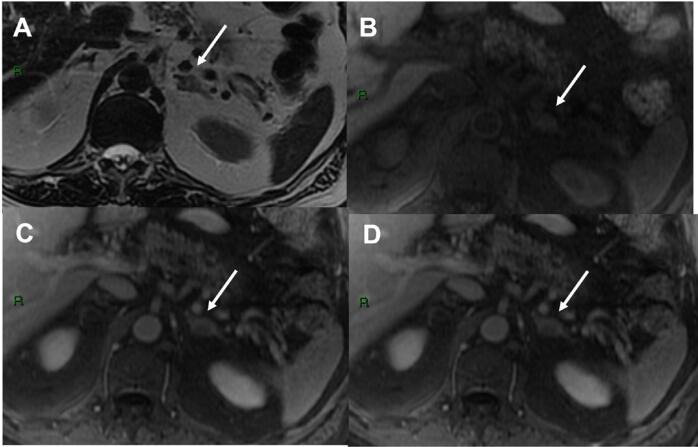
83-year-old man with oncology history of melanoma. Axial images show a 1.5 cm hyperintense heterogeneous lesion on T2-weighted (a) with no evidence of microscopic fat in in and out-of-phase images (not show). T1- weighted pre-contrast (b), portal (c) and delayed (d) post-contrast showed an absolute washout estimated at 7%, not suggestive of adenoma. This lesion was new relative to the anterior exam, had uptake on positron emission tomography (PET) and decreased in size after treatment (not show), compatible with metastasis

Age and sex comparison among the three groups had no statistically significant differences (p value 0.353 and 0.982, respectively). Oncologic history and signal on T2-weighted imaging differences were statistically significant among groups, showing a tendency of lesions being more heterogeneous and having hypersignal on T2-weighted imaging on non-adenoma group (Figure-1) greater than in the non-typical adenoma and typical adenomas (Table-3).

**Table 3:** Oncologic history and T2-weighted imaging analysis evaluated by groups.

Variable		Typical adenomas N (%)	Lipid poor adenomas N (%)	Non-adenomas N (%)	Total N (%)	p-value
**Oncologic history**						<0.001
	No	88 (89.8)	12 (80.0)	3 (30.)	103 (83.7)	
	Yes	10 (10.2)	3 (20.0)	7 (70.0)	20 (16.3)	
**T2 signal**						<0.001
	Homogeneous	91 (92.9)	10 (66.7)	2 (20.0)	103 (83.7)	
	Heterogeneous	7 (7.1)	5 (33.3)	8 (80.0)	20 (16.3)	
**T2 signal**						<0.001
	Hyposignal	94 (95.9)	10 (66.7)	5 (50.0)	109 (88.6)	
	Hypersignal	4 (4.1)	5 (33.3)	5 (50.0)	14 (11.4)	
**Total**		**98 (100.0)**	**15 (100.0)**	**10 (100.0)**	**123 (100.0)**	

Chi-square test

**Figure 2:**
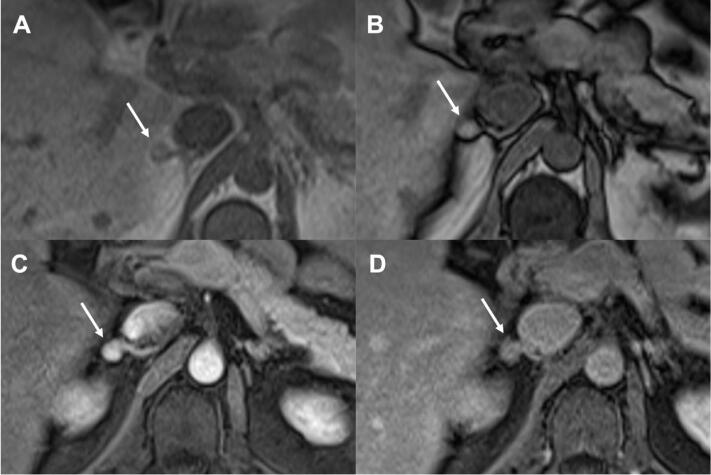
73-year-old woman with history of renal neoplasia and a typical adrenal adenoma. Axial images show a 1.2 nodule in the right adrenal gland with hyposignal on T2-weighted imaging (a), mild heterogeneous enhancement (b) and with presence of microscopic fat in in (a) and out-of-phase (b) images. Absolute washout was calculated at 66% and relative of 37%. The lesion remained was stable over 8 years.

**Figure 3:**
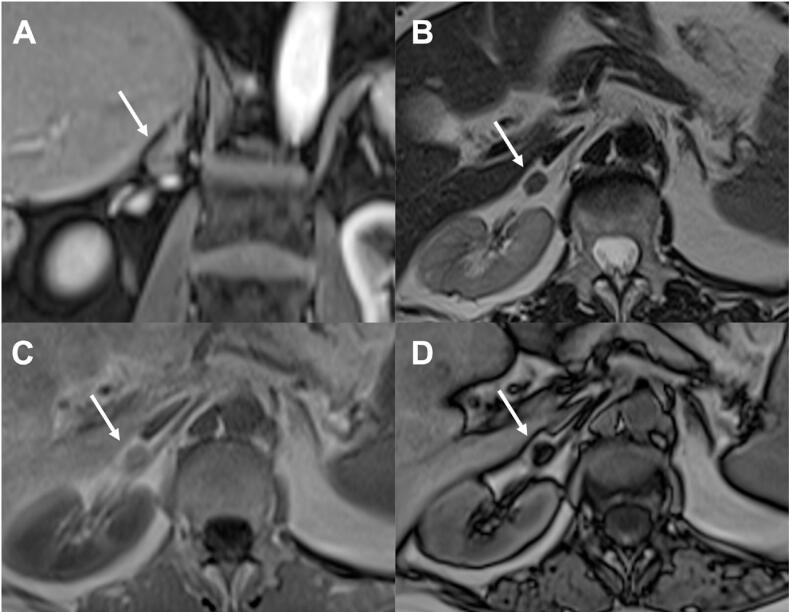
47-year-old man in investigation of a rectal lesion. Axial images show a 1.1 cm nodule in the right adrenal gland with no evidence of microscopic fat in in (a) and out-of-phase (b) images. The lesion was hypointense lesion on T2-weighted (not show), with hypervascular enhancement (c) and washout on delayed imaging (d). Absolute washout was calculated at 71% and relative of 35%. The lesion was stable over 3 years and the diagnosis of lipid-poor adenoma was made.

Size comparisons of adenoma vs. non-adenomas and lipid-poor adenomas vs. non-adenomas showed statistically significant differences (p value 0.004 and 0.002, respectively) (Figures 2 and 3).

In the typical adenoma group, 76 of the 98 (77%) lesions were included in curve pattern type 3, 19 (19%) in curve pattern type 2, and three (4%) in curve pattern type 1. Mean absolute and relative washouts were 29% and 16%, respectively. When performing a subgroup analysis, 70 lesions were evaluated on upper abdomen MRI protocol and 28 on abdomen and pelvis protocols, with mean absolute and relative washout of 25.2% (0 to 65%) and 13.8% (0 to 33%) for upper abdomen and 37.2% (0 to 66%) and 20.9% (0 to 45%) for abdomen and pelvis protocols, respectively (p-value <0.0004).

Among lipid-poor adenomas, 8 out of 15 (53%) lesions were included in curve pattern type 3, four (27%) in curve pattern type 2, and three (20%) in curve pattern type 1. Mean absolute and relative washouts were 21% and 10%. When performing a subgroup analysis, eight nodules were evaluated on upper abdomen protocol and seven on abdomen and pelvis protocol, with mean absolute and relative washouts of 14.8% (0 to 42%) and 5.8% (0 to 18%) for upper abdomen, and 28.4% (0 to 71%) and 14.1% (0 to 35%) for abdomen and pelvis protocols, respectively (p-value 0.239 and 0.126, respectively).

Among non-adenomas, four of 10 (40%) lesions were included in curve pattern type 3, four (40%) in curve pattern type 2, and two (20%) in curve pattern type 1. Mean absolute and relative washout were 10% and 5%. When performing a subgroup analysis, four nodules were evaluated on upper abdomen and six on abdomen and pelvis protocols, with mean absolute and relative washouts of 9.7% (0 to 25%) and 4.7% (0 to 15%) for upper abdomen and 19.1% (0 to 36%) and 9% (0 to 15%) for abdomen and pelvis protocols, respectively (p-value 0.246 and 0.349, respectively).

**Figure 4:**
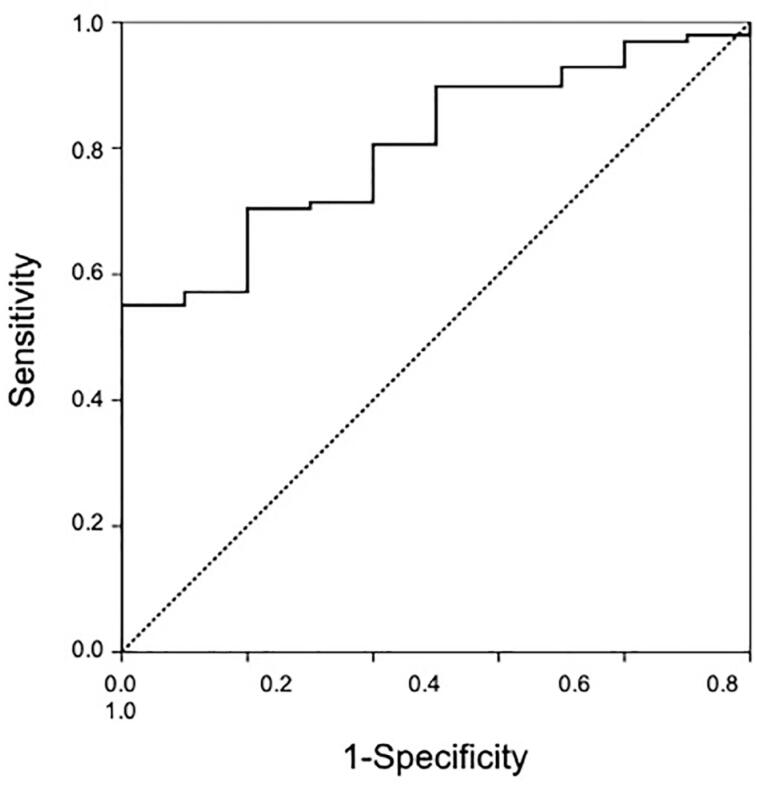
Receiver operating characteristic (ROC) curve comparing typical adenomas and non-adenomas groups with an area under the curve of 0.802.

When comparing the absolute and relative washouts among the groups, statistically significant differences were found in typical adenoma and non-adenoma groups, as displayed in Table-4. A receiver operating characteristic (ROC) curve analysis was carried out in this scenario; and using 11.75 as cut-off value, we obtained an area under the curve of 0.802, sensitivity of 71.4% and specificity of 70.0% (Figure-4).

**Table 4:** Comparisons of absolute and relative washouts among the three groups.

		Z-value	P-value
**Absolute washout**		
	Adenomas vs lipid-poor adenomas	1.20	0.229
	Adenomas vs non-adenomas	1.82	0.069
	Lipid-poor adenomas vs non-adenomas	0.66	0.508
**Relative washout**			
	Adenomas vs lipid-poor adenomas	1.90	0.058
	Adenomas vs non-adenomas	2.20	0.028
	Lipid-poor adenomas vs non-adenomas	0.50	0.618

## DISCUSSION

Adrenal nodules are frequent incidental findings in radiology daily routine, and lipid-rich adenomas are the most prevalent lesions. Most adenomas are well characterized on unenhanced CT with a definitive diagnosis and no additional study required. However, lipid-poor adenomas may occur in 30% of cases (16), and may not be fully characterized on imaging, even on CSI MRI. These lesions are considered indeterminate and pose management dilemmas (17). It is important to bear in mind that some non-adenoma lesions may display a high lipid content, such as extra-adrenal primary tumors producing lipid-rich adrenal metastases and be a potential analysis pitfall.

In cases of indeterminate nodules on CSI sequence, enhancement evaluation can aid differentiating benign and malignant lesions. Previous reports have suggested delayed contrast-enhanced MRI may be helpful in distinguishing adrenal tumors, since loss of signal intensity (SI) tends to be greater in adenomas than in non-adenomas, with a characteristic pattern of early enhancement and quick washout (18). The enhancement pattern of an arterial blush highly correlates with adenoma, even if there is negligible SI loss on out-of-phase images. Malignant lesions present an absence of capillary blush on immediate contrast-enhanced images and a prolonged retention in the tumor, due to tumor infiltration and loss of cellular membrane integrity. Rodacki et al. demonstrated adenomas have the greatest early enhancement on signal-time curve, persisting through the first 60 seconds, whereas metastases and pheochromocytomas showed maximum enhancement in the interstitial (delayed) phase (9).

Enhancement pattern analysis of adrenal nodules has been well described on CT. However, this protocol involves more radiation dose and a time consuming 15-minute delayed phase. Some reports have also evaluated a 10-minute (19) and 5-minute delay (20), both with good results. Therefore, the idea of a 3-5 minutes washout seemed promising on MRI. Our first analyses of enhancement curve patterns were not useful to differentiate non-typical adenomas and non-adenomas.

Total abdomen protocol yielded greater washout values than upper abdomen protocol, which is in agreement with previous literature on CT (8). Interestingly, even with a short 3-minute delay delayed post-contrast phase, few adenomas exhibited washout values higher than non-adenomas. High sensitivity and specificity were achieved when using absolute washout to differentiate typical adenomas from non-adenoma lesions.

Another important point of this study, despite not being the primary objective, was to observe differences in size, oncologic history, and signal on T2-weighted imaging had all statistical differences among groups, and these variables should be considered when an adrenal lesion is diagnosed in clinical practice. Increasing size in follow-up exams is not always related to malignancies (21) and a misdiagnosis of a benign lesion can be emotionally and financially harmful.

There are some limitations of this study. First, the majority of adrenal nodules were excluded due to no or shorter-than-2-year follow-up. Second, a homogeneous lesion stable over a 2-year follow-up period may not be an adenoma, but rather an indolent lesion. Third, we have not evaluated laboratory findings to identify functionality of the nodules. And finally, the exams were evaluated by a single experienced radiologist; hence an interobserver agreement could not be calculated.

## CONCLUSIONS

Enhancement pattern analysis and calculated washout may aid in the identification of lipid-poor adenomas on MRI. Those findings might be used to tailor more conservative approaches.
